# Extracellular matrix stiffness in hepatocellular carcinoma: mechanisms and targeted therapeutic strategies

**DOI:** 10.3389/fimmu.2026.1773909

**Published:** 2026-03-10

**Authors:** Zhuolin Zhou, Ximing Xu

**Affiliations:** Cancer Center, Wuhan University Renmin Hospital, Wuhan, China

**Keywords:** extracellular matrix stiffness, hepatocellular carcinoma, immunotherapy, pharmacological interventions, tumor microenvironment

## Abstract

Given the abundant stroma of the liver and that cirrhosis or hepatic fibrosis is the premalignant condition of most hepatocellular carcinomas (HCC), underscores the critical interaction between extracellular matrix (ECM) stiffness and the tumor microenvironment (TME) in the initiation, progression, and immunotherapy of HCC. This review presents a comprehensive exploration of the factors that regulate matrix stiffness, including the activation of cancer-associated fibroblasts (CAFs), the excessive deposition of ECM proteins, and cross-linking. Furthermore, this review explores the underlying molecular pathways through which matrix stiffness affects the prevalence of tumors and immune cells. Based on these premises, we delve into the potential targets and roles of pharmacological interventions targeting matrix stiffness in HCC and its immunotherapy, and highlight the considerable potential of biomaterials for the development of ECM stiffness-targeted agents. The potential exists for such agents to enhance the efficacy of immunotherapy and prolong the survival of patients diagnosed with HCC.

## 1 Introduction

Hepatocellular carcinoma (HCC) is one of the most prevalent malignant tumors of the gastrointestinal tract globally, and the third leading cause of cancer-related mortality (1). This unsatisfactory outcome is primarily attributable to the low rate of early diagnosis and the proclivity of HCC patients to recurrent metastasis (2). Although cancer therapeutic strategies have advanced considerably in recent years, including tumor ablation, molecular targeted therapy, and immune checkpoint inhibitors (ICIs), HCC is a highly heterogeneous tumor that exhibits inferior therapeutic outcomes relative to other cancer types, with the 5-year survival rate of advanced HCC at merely 21% (3). Sorafenib and lenvatinib are tyrosine kinase inhibitors (TKIs) approved for the first-line treatment of HCC, yet most patients with HCC eventually develop resistance (4). The IMbrave150 global multicenter Phase III study demonstrated that atezolizumab combined with bevacizumab yielded a superior survival benefit relative to sorafenib monotherapy (5). However, the combination of atezolizumab and cabozantinib failed to confer a favorable clinical benefit, indicating that the molecular mechanisms driving drug resistance and the efficacy of immunotherapeutic combinations in HCC remain unclear (6).

The extracellular matrix (ECM) is primarily composed of a range of proteins and glycosaminoglycans (GAGs), and plays a crucial role within the tumor microenvironment (TME). The ECM provides structural and biochemical support for the regulation of cell and tissue growth by sequestering cytokines and growth factors (7). It is widely postulated that cirrhosis or hepatic fibrosis constitutes the initial stage in HCC development. During this process, stromal cell activation and dysregulation of ECM components result in increased liver stiffness. Furthermore, matrix stiffness changes dynamically with the pathological process, converting mechanophysical signals into various biochemical responses. Ultimately, this drives the progression of cirrhosis and subsequent malignant transformation to HCC (8). The present study hypothesises that matrix stiffness can serve as a valid predictor of HCC (9). Furthermore, the role of matrix stiffness in the immune evasion of tumor cells warrants further investigation. For instance, tumor cells induce the formation of an immunosuppressive barrier by elevating ECM stiffness, thereby impeding immune cell infiltration into the TME and compromising the efficacy of tumor-targeted ICIs (10). A more in-depth understanding of how matrix stiffness modulates HCC progression and immunotherapy is therefore imperative for optimizing clinical treatment strategies.

The objective of this review is to summarize recent advances in the study of matrix stiffness in HCC and to explore the mechanisms by which matrix stiffness modulates HCC progression and immune cell function. In order to construct a cohesive narrative for the present review, an exhaustive search of the English language literature was conducted across four databases, including PubMed, Web of Science, Embase, and Scopus, encompassing publications up to November 30, 2025. This search strategy combined all possible key terms such as “extracellular matrix stiffness, “ “matrix rigidity, “ “mechanobiology, “ “mechanotransduction, “ “hepatocellular carcinoma, “ and “liver cancer, “ along with medical Subject headings (MeSH) terms. Literature selection was guided by thematic relevance to ECM stiffness in HCC, prioritizing primary research and high-quality reviews that directly address its role in tumor progression, therapy resistance, and immune regulation, while also including seminal earlier works for necessary context. In light of the aforementioned findings, the potential therapeutic benefits that arise from targeting matrix stiffness-related targets in HCC and immunotherapy are a subject of extensive discussion. Overall, this review underscores the significance of matrix stiffness in HCC, which is anticipated to yield novel therapeutic approaches.

## 2 Factors regulating matrix stiffness in the liver

**Figure 1:**
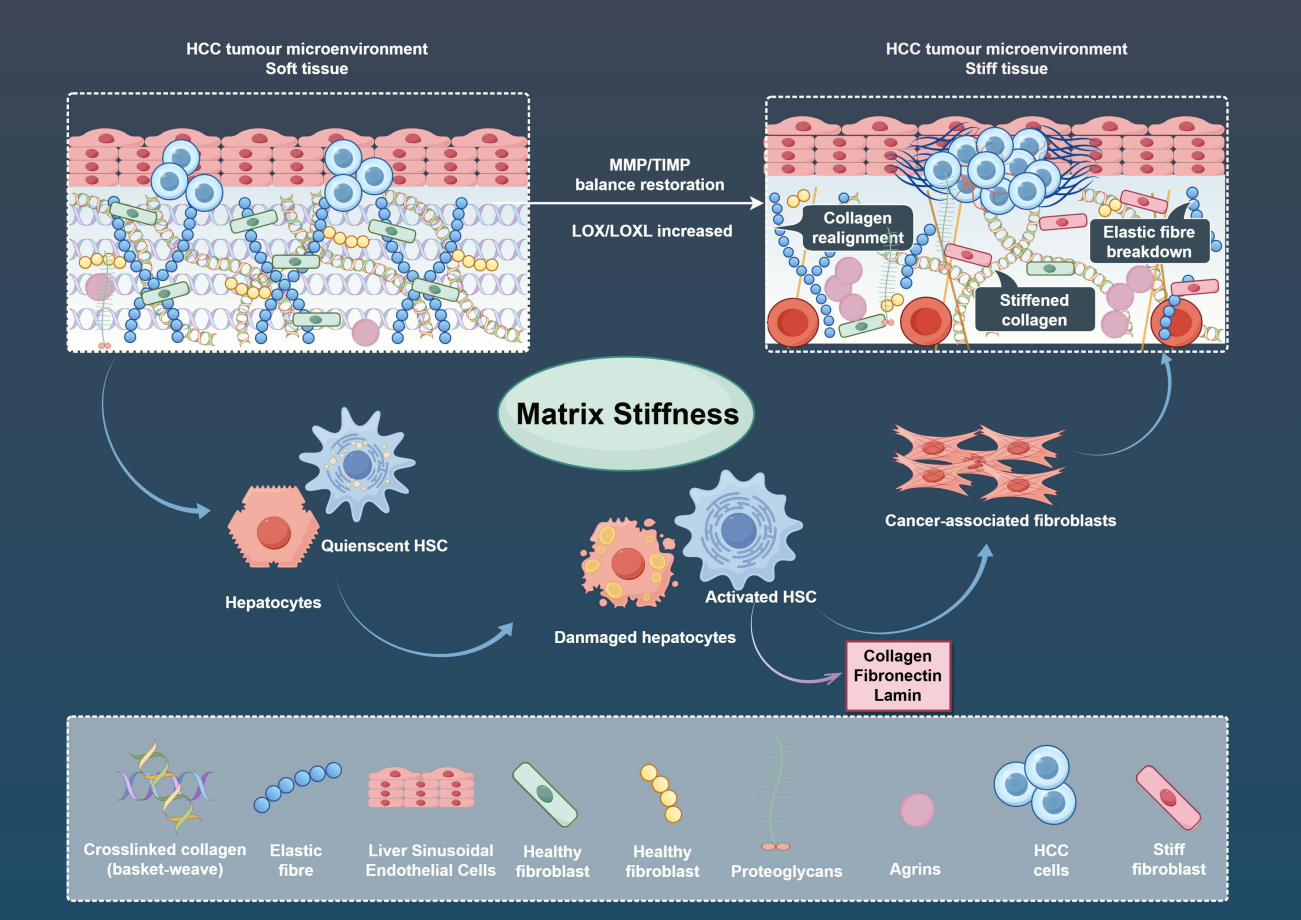
The status of different matrix stiffness levels in hepatocellular carcinoma (HCC) and their effects on HCC-associated cells. HSC, hepatic stellate cells; MMP, matrix metalloproteinase; TIMP, tissue inhibitor of metalloproteinases; LOX, Lysyl Oxidase; LOXL, Lysyl Oxidase-like.

Current evidence indicates that elevated matrix stiffness is predominantly driven by ECM remodeling. As illustrated in **Figure 1**, the process is primarily regulated by several key factors, including the abnormal deposition of ECM components such as collagen, laminin, elastin, matrix metalloproteinase (MMP)/tissue inhibitor of metalloproteinases (TIMP), and collagen cross-linking.

The abnormal deposition of various ECM components and increasing matrix stiffness are important features in the development of HCC. During hepatic fibrosis, chronic inflammatory stimuli (e.g., viral infection, alcoholic injury, and hepatic steatosis) activate quiescent hepatic stellate cells (HSCs) and other mesenchymal cells, driving their differentiation into myofibroblasts. These activated cells secrete large quantities of matrix proteins, including collagen, fibronectin (Fn), and laminin, which promotes aberrant ECM deposition and subsequent elevation of matrix stiffness (11–14). During hepatic ECM remodeling, laminin and type IV collagen are replaced by interstitial collagens (types I and III) (15). Among these, type I collagen is the most abundant collagen isoform linked to cirrhosis and is strongly correlated with matrix stiffness (16). MyD88 inhibition in HSCs reduces type I collagen, thereby attenuating hepatic fibrosis (17). Hepatic parenchymal cells have also been shown to increase tissue stiffness via FHL2 upregulation and enhanced matrix protein expression (18). Targeting TXNDC5 in fibrotic lesions inhibits the activation of HSCs for non-classical transforming growth factor β (TGFβ) signalling to attenuate hepatic fibrosis, suggesting that fibrosis induced by HSCs activation is reversible (19). Rho-associated kinase (ROCK), a identified mechanosensor of matrix stiffness, regulates matrix protein synthesis to further elevate stiffness (20). Matrix stiffness additionally promotes ROCK-mediated AKT signalling activation, exacerbating hepatic fibrosis and forming a self-perpetuating feedback loop (21). In contrast, FN was found to exert a negative regulatory effect on hepatic fibrosis and matrix stiffness by inhibiting the activation of HSCs (22). These findings suggest the possibility of distinct regulatory effects of matrix proteins on matrix stiffness.

Proteoglycans (PGs), major ECM components composed of glycosaminoglycans (GAGs) bound to core proteins, correlate with matrix stiffness in liver tissue via excessive deposition (23). Syndecan-1 (SDC1) is a PG expressed primarily on the surface of hepatic cells. It is highly expressed in patients with cirrhosis and HCC, and its expression is positively correlated with the severity of hepatic fibrosis (24). Notably, SDC1 inhibits hepatic fibrosis by promoting early clearance of TGFβ1 and upregulating hepatic MMP14 expression (25), supporting the hypothesis that SDC1 may regulate hepatic fibrosis in a bidirectional manner. Agrin is a heparan sulfate PG that plays a key role in the ECM as a major secreted component of neuromuscular junctions (NMJs) (26). Consistent with the abnormal expression pattern of other PGs in liver diseases, Agrin is also overexpressed in cirrhosis and HCC, serving as a sensor in HCC for matrix stiffness mechanical signals (27). Yes-associated protein (YAP) and TAZ, a key molecule of the Hippo pathway, have been identified as key factors in the development of hepatic fibrosis and HCC. Through a process of nucleus entry and binding to the TEAD domain, with the process being widely recognized as a sensor for transducing mechanical signals of matrix stiffness (28, 29). Agrin enhances YAP-TEAD interaction via the LRP4-MuSK and the integrin-focal adhesion pathways to facilitate the process of ECM remodelling and increased matrix stiffness in HCC (30). In TME of HCC, tumor-secreted Agrin enhances the stability of vascular endothelial growth factor receptor 2 (VEGFR2) by increasing matrix stiffness, making HCC highly vascularized (31).

ECM degradation by enzymes is an additional factor contributing to matrix stiffness variability. MMP and its inhibitor (TIMP) maintain dynamic equilibrium to regulate hepatic ECM expression and deposition. The inhibition of MMP-1 secretion reverses extracellular bead protein-mediated hepatic fibrosis (32). MMP-9 mediates ECM degradation in liver sinusoidal endothelial cells (dLSECs) to attenuate advanced hepatic fibrosis in mice, showing strong collagen-degrading capacity (33). Moreover, an elevated level of MMP-2 expression was observed in high matrix stiffness conditions (34). Xie et al. found that modulating the MMP-2/TIMP-1 ratio may influence hepatic fibrosis progression (35), and multiple studies have highlighted the pivotal role of MMP/TIMP interactions in hepatic fibrosis (36, 37). Cross-linking is an important step in collagen synthesis, and its degree correlates closely with matrix stiffness mechanical properties. During cross-linking, the specific hydroxylation of collagen telopeptides is initiated by lysyl hydroxylase (LH). It was found that knockdown of 2-Oxoglutarate 5-Dioxygenase 2 (PLOD2), which encodes LH2, significantly increased collagen solubility and inhibited ECM matrix stiffness escalation (38). LH2-induced matrix stiffness elevation results from its ability to promote the transition of cellular matrix towards higher levels of hydroxylysine aldehyde-derived collagen cross-links (39). In addition, at the 3D collagen gel level, both LH1 and LH2 increased matrix stiffness by promoting collagen cross-linking (40, 41). Lysyl Oxidase (LOX) oxidises hydroxylated lysine and hydroxylysine to form residues that spontaneously condense with adjacent residues to form cross-linked collagen to increase matrix stiffness (42). The LOX family mainly includes LOX and LOX-like (LOXL1-4) proteins (43), and its expression is elevated in patients with hepatic fibrosis and HCC, correlating with poor prognosis (42, 44, 45). Of particular note is the central role of LOXL2 in insoluble collagen deposition during ECM remodelling, which is correlated with matrix stiffness (46). Activated HSCs secrete Periostin, a stromal cell glycoprotein that promotes collagen cross-linking by activating LOX and LOXL via pathways such as phosphorylating SMAD2/3 or binding BMP-1 (47, 48). On the other hand, Zhao et al. identified a strong correlation between LOXL1, elastin, and collagen I in a mouse model of hepatic fibrosis (49).

## 3 Effect of matrix stiffness on HCC-associated cells

### 3.1 Hepatic stellate cells and cancer-associated fibroblasts

HSCs are the main cellular source of myofibroblasts during liver ECM remodelling, distributed in the Disse space between liver sinusoidal endothelial cells (LSECs) and hepatocytes (50). High matrix stiffness can induce HSC activation via multiple signalling pathways to promote hepatic fibrosis and HCC progression, including ERK or PKA/AKT pathway activation, Ras homolog gene family member (RhoA) involvement, and enhanced exosome secretion (21, 51, 52). For instance, high matrix stiffness significantly activates HSCs by inducing integrin β1 activation and YAP nuclear translocation, and this phenomenon is reversible (13). Furthermore, mechanotransduction pathways can exacerbate HSCs’ migration and accumulation in high-stiffness regions, thus increasing tissue stiffness and forming a positive feedback loop (11).

Cancer-associated fibroblasts (CAFs) primarily originate from activated HSC differentiation and can feedback matrix stiffness mechanical signals to regulate cellular biochemical responses. Due to the high heterogeneity of CAFs, Wei et al. used RNA-seq to identify fibrosis markers collagen type I alpha 1 (COL1A1), collagen type III alpha 1 (COL3A1), and LOX as closely associated with CAF development. Notably, the YAP1-positive CAF subpopulation is a key contributor to HCC matrix stiffness and promotes HCC progression by activating signalling pathways such as autophagy through up-regulating matrix stiffness-related genes (53). In addition to the ECM component, the transformation process is influenced by TGF-β. In HCC TME, TGF-β secretion by HCC cells activates CAFs, leading to the production of cardiotrophin-like cytokine factor 1 (CLCF1), which in turn increases TGF-β secretion and accelerates HCC progression (54). It has been demonstrated that CAFs generate physical trajectories in the ECM through their remodelling properties and exertion of physical tensile forces, enabling collective cancer cell invasion. CAFs’ depletion reduces the ability of tumors to metastasise in the liver and this facilitation is mechanically limited by collagen I formation, suggesting that targeting CAFs might reduce tumor metastasis (55).

### 3.2 Hepatocytes

Hepatocytes, the principal parenchymal and functional liver cells, have high regenerative capacity and are sensitive to elevated matrix stiffness during liver injury (56). Damaged hepatocytes develop lipid accumulation, mitochondrial DNA damage, and oxidative stress disorders, leading to excessive production of reactive oxygen species (ROS) and further exacerbating hepatic fibrosis (57). During this process, the expression or secretion of fibrosis-related genes in hepatocytes is also altered, such as Notch (58), TAZ (59), and TGF-β (60). Additionally, high matrix stiffness participates in regulating various hepatocyte functions such as morphological characteristics, migration, and apoptosis (61).

### 3.3 Liver cancer stem cells

Liver cancer stem cells (LCSCs) are a subpopulation of tumor cells with self-renewal ability and differentiation potential in HCC, playing an important role in the recurrence and metastasis of HCC. Increased matrix stiffness resulted in the up-regulation of stemness genes, including CD133, EpCAM, and Nanog in HCC cells, indicating an association between the matrix stiffness-mediated mTOR signalling pathway and the regulation of HCC stemness (62). On the contrary, Tian et al. (63) concluded that LCSCs cultured on soft substrates showed more pronounced expression of stemness genes such as Oct-4, Sox-2, CXCR4, and CD133, increased side population cell numbers, and facilitated tumor cell adaptation to environmental changes. Similar conclusions were obtained by Ng et al. (64). CD133-positive LCSCs enhance and maintain their own stemness and drug resistance by remodelling the ECM and creating a microenvironment of local soft matrix stiffness around themselves. The divergent outcomes of the aforementioned studies may be attributable to variations in the type of matrix material, porosity, and viscoelasticity employed, in addition to the heterogeneity of the physical microenvironment. Consequently, the impact of matrix stiffness on the stemness of LCSCs remains ambiguous.

## 4 Effect of matrix stiffness on HCC

**Figure 2:**
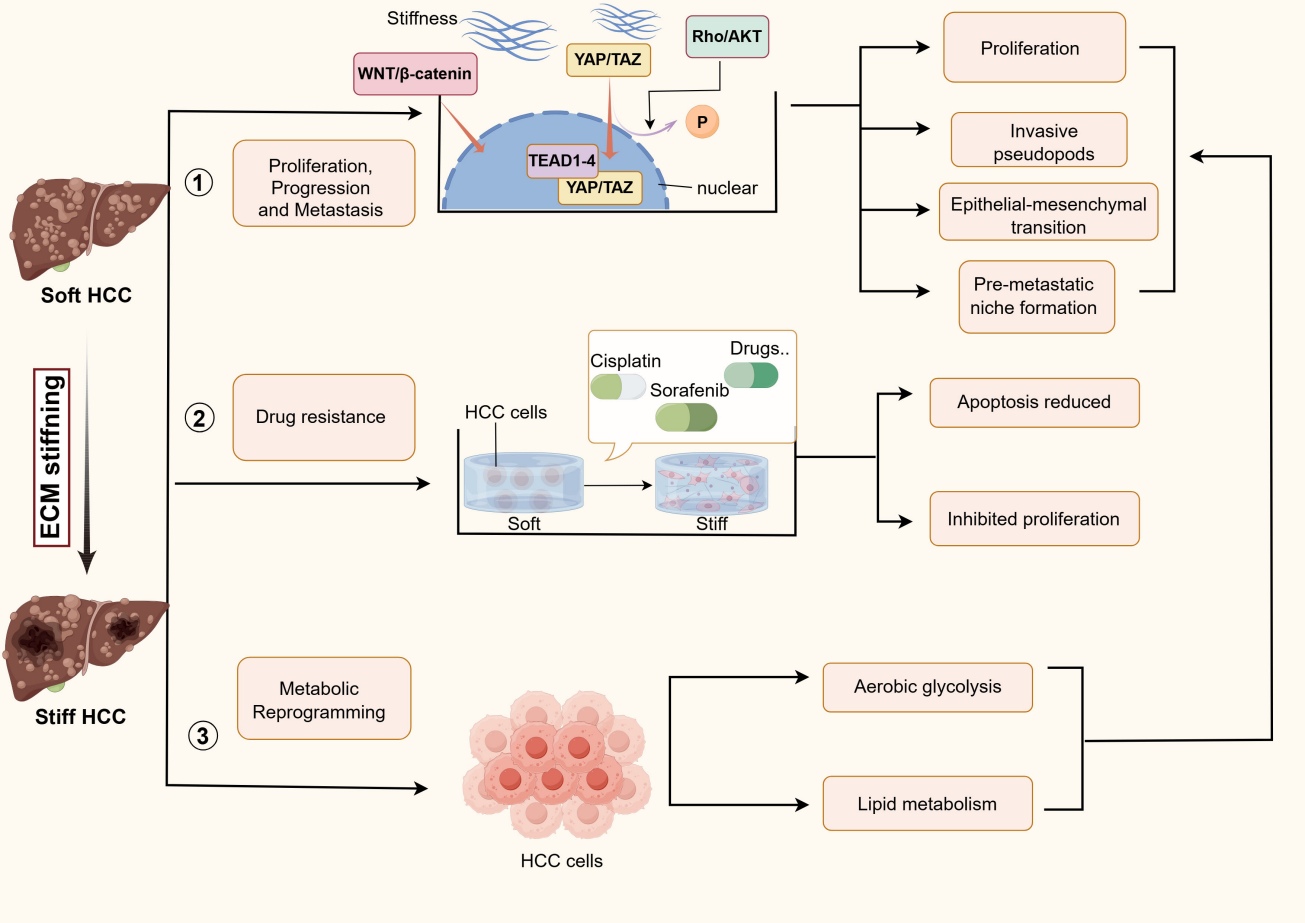
The impact of matrix stiffness on the biological behaviors of hepatocellular carcinoma (HCC). YAP, yes-associated protein; TAZ, transcriptional coactivator with PDZ-binding motif; TEAD, transcriptional enhanced associate domain; WNT, wingless/integrated.

In the context of liver biology, matrix stiffness is defined as a biophysical property of the tissue that quantifies its resistance to deformation under an applied force. It is commonly measured as the elastic modulus (in kilopascals, kPa) and reflects the combined mechanical contributions of ECM components. In a healthy liver, the ECM provides a compliant and dynamically balanced scaffold that supports the functions of parenchymal and non-parenchymal cells, with a characteristic stiffness of approximately 0.2 to 1 kPa. During the progression of chronic liver disease (e.g., fibrosis and cirrhosis), excessive deposition, aberrant remodeling, and cross-linking of ECM proteins, notably collagen type I, lead to a pathologically increased matrix stiffness, which can escalate from 2-6 kPa in fibrosis to over 20 kPa in advanced cirrhosis and HCC (65, 66). This elevated stiffness is not just a passive structural change but an active biomechanical cue that dysregulates cellular behaviour via mechanotransduction pathways, driving disease progression. Thus, exploring matrix stiffness in HCC development and progression is critical (**Figure 2**).

### 4.1 Effect of proliferation, progression, and metastasis of HCC

Growing evidence indicates that increased matrix stiffness is involved in the onset, progression, metastasis, and other malignant processes of HCC. It was found that the proliferation of HCC cells in high matrix stiffness culture was significantly increased (10). Bioinformatics analysis revealed a close association between matrix stiffness-related ECM proteins and HCC prognosis, and further *in vitro* and *in vivo* experiments validated that the matrix stiffness-related protein DYNLL1 promotes HCC cell progression and migration via the Wnt/β-catenin pathway (67). Matrix stiffness activates USP40 transcription via the YAP, thereby promoting YAP nuclear accumulation to facilitate HCC progression (68). It also induces KAT6A, which drives HCC progression through upregulating the SOX2 pathway (69). Moreover, increased matrix stiffness activates the RhoA-AKT axis, inducing P300 nuclear translocation and enhancing HCC cell growth (21, 70). The role of exosomes in influencing the tumor microenvironment and promoting tumorigenesis and metastasis has been extensively studied (71). Wu et al. found that rigid matrix activates Rab8 through activation of AKT, which increases exosome secretion in HCC cells cultured under high matrix stiffness. These exosomes synergistically interact with matrix stiffness to further activate the Notch pathway, promoting tumor growth and invasion (72). On the other hand, matrix stiffness activates CAFs induced by CD36-AKT-E2F3 signalling, enhancing HCC growth and metastasis (73). Anna et al. found that matrix stiffness may induce autophagy in fibroblasts or stellate cells by regulating the stabilisation of AMPKα at adhesion patches, thereby promoting the growth of neighbouring tumor cells (74). The hypothesis that matrix stiffness may promote tumor growth by co-regulating tumor and stromal cells merits further investigation.

On higher matrix stiffness, HCC cells typically display an elongated, flattened and more spread morphology, accompanied by distinct pseudopod formation (9). The formation of invasive pseudopods is a key hallmark of cancer and closely associated with cancer cell invasion, metastasis and other biological behaviours (75). Zhang et al. demonstrated that increased matrix stiffness mainly enhances HCC migration and invasion by activating the integrin β1 receptor or inducing epidermal growth factor (EGF) production, which directly or indirectly triggers invasive pseudopodia formation via the EGFR/Src/Arg/cortactin pathway (76). Epithelial-mesenchymal transition (EMT) frequently occurs during tumor invasion and metastasis. Investigation revealed that matrix stiffness can independently induce EMT in HCC cells; in addition to SNAIL, the C-X-C chemokine receptor type 4 (CXCR4) has also been demonstrated to mediate the YAP signalling pathway and is associated with poor outcomes in patients diagnosed with HCC (52, 77). Moreover, cancer cell intravasation into the bloodstream is an essential step during cancer metastasis. The findings indicate that matrix stiffness-induced upregulation of Piezo1 and inhibition of integrin β1 and αVβ5 mediate elevated vascular endothelial growth factor (VEGF) expression in HCC and promote tumor angiogenesis (78, 79). As a highly metastatic neoplasm, advanced HCC is frequently accompanied by multiple organ metastases in patients. Increased matrix stiffness significantly up-regulated LOXL2 expression in HCC cells and promoted the up-regulation of FN, MMP9, CXCL12 expression, and BMDC recruitment to assist in the formation of pre-metastatic niche formation in HCC (80, 81). Furthermore, it was found that elevated lung matrix stiffness induced pre-metastatic niche formation during HCC lung metastasis (82). Nevertheless, further studies are warranted to elucidate the mechanisms by which matrix stiffness within primary HCC tumors regulates pre-metastatic niche formation.

### 4.2 Effect of drug resistance in HCC

Drug therapy often leads to drug resistance in HCC patients due to factors such as cellular differentiation, immune infiltration, and changes in the composition of the ECM. It was demonstrated that matrix stiffness contributes to the induction of drug resistance in cancer cells (83). *In vitro* experiments showed that HCC cells cultured under high matrix stiffness conditions after cisplatin treatment exhibited reduced apoptosis and augmented cisplatin resistance (9). HCC cells displayed divergent morphologies when cultured on high matrix stiffness hydrogels and showed attenuated sorafenib-induced apoptosis (84). Increasing evidence indicates that matrix stiffness synergistically inhibits HCC ferroptosis together with ferroptosis-related genes, thereby contributing to sorafenib resistance in HCC patients (85, 86). 3D culture levels are more reliable than 2D culture conditions for mimicking the *in vivo* tissue environment. The use of alginate gel (ALG) beads to simulate 3D matrix stiffness was shown to induce responses in HCC cells, characterized by inhibited proliferation and increased resistance to paclitaxel, 5-FU, and cisplatin under elevated matrix stiffness conditions (87). This phenomenon may be attributed to the preferential targeting of chemotherapeutic agents to rapidly proliferating tumor cells. Özkan A. et al. found that HCC cells cultured on 3D collagen I gels of varying stiffness exhibited significantly greater resistance to adriamycin and sorafenib, with more pronounced chemoresistance (88). These findings suggest that future clinically targeted drug combinations targeting matrix stiffness may prove effective in reducing chemotherapy resistance in patients.

### 4.3 Effect on metabolic reprogramming of HCC cells

The rapid proliferation of malignant tumors is inseparable from adjustments in tumor energy metabolism, which is considered to be one of the hallmarks of cancer (89). The Warburg effect describes the metabolic reprogramming by which highly proliferative tumor cells sustain energy production via aerobic glycolysis for ATP generation, and beyond this, tumor cell metabolic reprogramming further encompasses glutamine metabolism, lipid metabolism, macromolecular synthesis, and redox homeostasis (90). Recent studies have indicated that metformin holds potential efficacy in treating HCC (91–93). Metformin counteracts the Warburg effect by activating adenosine 5’-monophosphate (AMP)-activated protein kinase (AMPK), which can effectively inhibit oxidative phosphorylation in tumor cells (94). However, the inhibitory effect of metformin on HCC invasion and metastasis is abrogated by downstream signalling pathways activated by elevated matrix stiffness (95). Furthermore, matrix stiffness exerts a synergistic effect with tumor metabolic reprogramming. To illustrate this point, matrix stiffness activated YAP into the nucleus and significantly enhanced aerobic glycolysis and promoted tumor migration in HCC cells (96). SCD1 was responsive to elevated matrix stiffness and promoted HCC progression by reprogramming lipid metabolism in HCC cells (97). Matrix stiffness promoted HCC progression by directly regulating lipid metabolism via modulation of long-chain acyl-CoA dehydrogenase (ACADL) (98). These aforementioned findings provide evidence for a complex interplay between the mechanical transduction of matrix stiffness within the TME and cancer metabolic reprogramming, a relationship that warrants further in-depth investigation into the underlying mechanisms.

## 5 Role of matrix stiffness in immunotherapy of HCC

**Figure 3:**
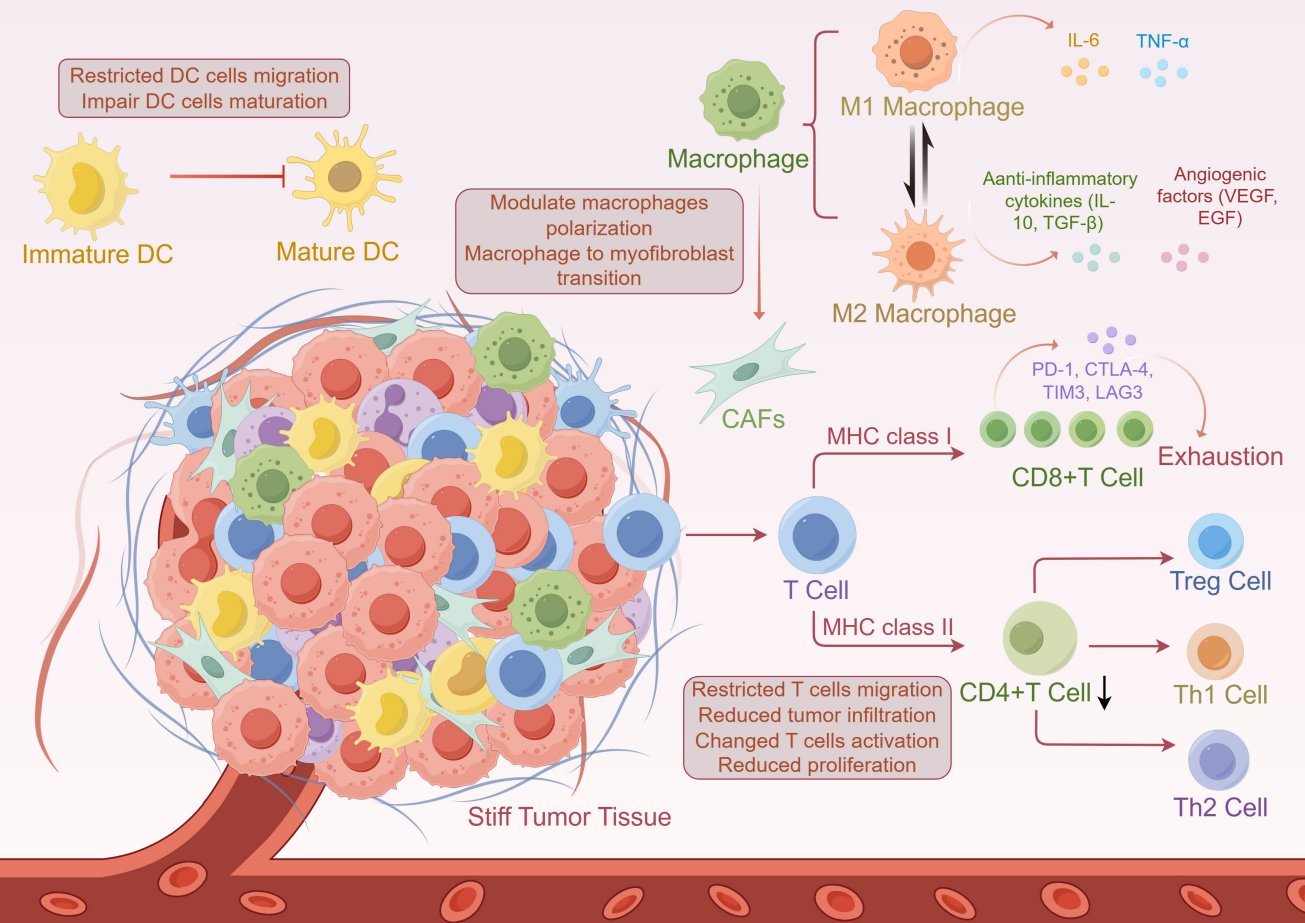
The impact of matrix stiffness on the immune microenvironment of hepatocellular carcinoma (HCC). Treg, regulatory T cell; DC, dendritic cell; CAFs, cancer-associated fibroblasts; MHC, major histocompatibility complex; TNF-α, tumor necrosis factor-α; TGF-β, transforming growth factor-β; VEGF, vascular endothelial growth factor; EGF, epidermal growth factor.

Tumors have the capacity to evade immune system surveillance, recognition, and clearance through various mechanisms. As tumors progress, they recruit inflammatory immune cells, which gradually shifts the immune profile of the TME from anti-tumor to tumor-promoting (99). Immune cells in TME mainly include dendritic cells (DCs), cytotoxic T cells (CTLs), regulatory T cells (Tregs), tumor-associated macrophages (TAMs), natural killer cells (NK), etc. The prognosis of tumors and the response to immunopharmaceuticals such as ICIs are closely related to the spatial distribution of immune cells in the TME and surrounding stroma (100, 101). In addition, the complexity of the TME is reflected in how immune cells alter their function in response to changes in the physical properties of the tissue microenvironment, referred to as mechanoimmunology (102). A comprehensive understanding of how matrix stiffness impacts immune cells and their functionality within the TME is imperative to enhance the effectiveness of HCC immunotherapy (**Figure 3**).

### 5.1 Effect of matrix stiffness on T cells

T cells are lymphoid-like cells that are classified into distinct subpopulations based on their recognition of peptide antigens and surface glycoproteins, and serve as a key component in investigating tumor immunotherapy heterogeneity. In this field, the most extensively studied and clinically significant subpopulations are CD4+ and CD8+ T cells. CD4+ T cells are referred to as “helper T cells” due to their role in coordinating immune cell function by activating memory B cells and CD8+ T cells. CD8+ T cells, also known as CTLs, are critical for tumor immunotherapy, as they can directly target and eliminate tumor cells via their cytotoxic activity.

As shown in previous studies, modifications in matrix stiffness can impede T cell migration and infiltration into the TME by forming an additional matrix barrier (103). A study analyzing RNA-seq data from HCC revealed that ECM-related genes were significantly upregulated in CD8+ T cell-excluded peritumoral tumors, suggesting that tumor fibrosis influences tumor immune evasion (104). Moreover, increased matrix stiffness is closely associated with changes in T cell function. For example, soft matrix stiffness increased T cell proliferation, while T cells cultured on stiffer matrices exhibited diminished activation (105). Conversely, Judokusumo et al. found that naive CD4+ T cells exhibited heightened activation in response to increasing stiffness, as evidenced by elevated IL-2 secretion (106). Yuan et al. discovered that proliferation and IL-2 secretion of primary human-derived T cells exhibited a biphasic functional response to matrix stiffness, correlating with the density of T cell-activating antibodies (107). This may explain why previous studies have reached such divergent conclusions. During ECM remodelling, vascular calcification and metabolic reprogramming may deprive T cells of the energy and nutrients needed for their function and differentiation (108).

The mechanosignalling sensor Piezo1 is strongly associated with cancer immunity (109). However, Piezo1 deletion in T cells is not necessary for T cell activation, but can affect increasing Tregs (110). A further study established that matrix stiffness can influence the differentiation balance between Th1 cells and Tregs via Piezo1 (111). Concurrently, matrix stiffness regulates the epigenetic modification of Treg-specific genes and the suppressive function of Tregs (112). T-cell exhaustion (TEX) is a differentiated state in which CD8+ T cells have impaired cytotoxicity due to prolonged stimulation by cancer antigens in the TME. Peng et al. found that collagen interaction with CD18 via LAIR1 induces CD8+ TEX, leading to impaired CD8+ T cytotoxicity (113). Conversely, exhausted T cells exhibited augmented expression of inhibitory receptors, including PD-1, CTLA-4, TIM3, LAG3, and other genes closely linked to immune evasion (114, 115). Increased matrix stiffness in HCC transduces mechanical signals mediated by the Piezo1/calcium/CREB axis to activate the transcription factor transcription factor (TF) Odd-skipped related 2 (Osr2), which significantly contributes to CD8+ T cell exhaustion in the TME and impairs the efficacy of immunotherapy in patients with HCC (116).

### 5.2 Effect of matrix stiffness on macrophages

Macrophages are innate immune cells that originate in the bone marrow and differentiate from monocytes to mature macrophages after infiltrating tissues and organs. Classically activated macrophages, referred to as M1 or pro-inflammatory macrophages, secrete pro-inflammatory cytokines such as tumor necrosis factor-α (TNF-α), IL-1β, and IL-6. Alternatively, their alternative activation phenotype yields M2 macrophages, also referred to as anti-inflammatory macrophages, which promote tumor formation and progression by secreting matrix-degrading enzymes, angiogenic factors, and anti-inflammatory cytokines (117).

Macrophages are recognized as key drivers of fibrogenesis. However, the regulatory role of macrophages in hepatic fibrosis is complex, which underlies the contradictory findings reported across multiple studies (118). Piezo1 transduces mechanical signals to macrophages, while its activity is also involved in regulating macrophage polarisation responses (109). A study found that knockdown of Piezo1 reduced hepatic inflammation by decreasing macrophage infiltration and M1 polarisation, and attenuated hepatic fibrosis by reducing collagen deposition (119). In contrast, Wang et al. proposed that the Piezo1 activation promotes stiffness-dependent cytotoxicity in macrophages to present pathogen antigens during hepatic fibrosis, thereby accelerating the regression of inflammation and fibrosis (120). In addition, macrophages on soft matrix substrates exhibited increased ROS secretion, which in turn activated the NF-κB pathway and drove their M1 polarization, whereas those on moderately stiff matrices underwent M2 polarization (121). For instance, an investigation into HCC tissues on various matrix stiffnesses revealed that increased matrix stiffness promotes M2 macrophage polarization (122). Defects in the nuclear membrane protein SUN1/2 in macrophages regulate M1 polarization by functioning as mechanoregulators to induce nuclear contraction and softening (123). Liu et al. found that the biomimetic matrix could induce the M1 polarisation of macrophages through multiple mechanical feedback modes by using a biopolymer membrane to mimic the matrix stiffness changes in HCC (124). The hypothesis is proposed that macrophages bidirectionally regulate the immune response in response to stiff microenvironmental changes (125). The variation in conclusions across different experiments can be attributed to the use of distinct materials and culture methods. In conclusion, macrophages exhibit sensitivity to matrix stiffness and function as ‘central regulators’ of hepatic fibrosis.

Recent findings indicate that macrophages have the potential to undergo a transformation into myofibroblasts, resulting in fibrosis in various organs, including the heart, kidneys, lungs, and liver. This process is referred to as the macrophage-to-myofibroblast transition (MMT) (126–129). MMT cells are characterised by co-expression of myofibroblast markers (collagen I or α-SMA) with macrophage markers (F4/80 or CD68) (130). In a given study, MMT was proposed as a novel mechanism through which TAM promotes the formation of CAFs (131). Inhibition of the MMT-specific regulator RUNX1 effectively inhibits MMT-driven CAFs and tumor formation *in vivo*, providing a novel approach for the development of future clinical cancer therapies (132). However, the study of MMT in cancer is still very limited, and related studies in HCC have not yet been reported, suggesting that further in-depth studies are needed for researchers to investigate the correlative mechanisms that exist between macrophages and matrix stiffness.

### 5.3 Impact of matrix stiffness on antigen presentation mechanisms

The liver occupies a unique position within the immune system, acting as both a metabolic hub and an immune organ with a dominant tolerogenic phenotype. This dual role is particularly critical in the context of chronic liver disease and liver fibrosis progressing to HCC. Hepatocytes, liver sinusoidal endothelial cells, Kupffer cells, DCs, and HSCs all contribute, directly or indirectly, to antigen presentation through major histocompatibility complex (MHC) class I and class II pathways (133). These processes are tightly regulated under physiological conditions to prevent excessive immune activation in response to gut-derived antigens and endogenous metabolic by-products. However, progressive extracellular matrix remodeling and increased tissue stiffness profoundly alter this immunological balance.

In hepatocytes, MHC class I expression is constitutive but typically maintained at relatively low levels compared with professional antigen-presenting cells (134). Chronic inflammation, interferon signaling, and endoplasmic reticulum stress can upregulate class I antigen presentation machinery, enhancing the display of tumor-associated or neoantigenic peptides to CD8+ T cells (135). As outlined previously, matrix stiffness modulates intracellular stress pathways (121), mechanosensitive transcription factors (68), and metabolic reprogramming (98), all of which intersect with antigen processing pathways (including proteasomal degradation and peptide loading), thereby indirectly regulating this MHC class I antigen presentation process. Sustained mechanical stress has been shown in other epithelial systems to alter proteostasis and unfolded protein responses, raising the possibility that stiff fibrotic matrices reshape the antigenic landscape presented by hepatocytes in chronic liver disease and hepatocellular carcinoma (136). In contrast, MHC class II expression in the liver is more restricted and typically associated with professional antigen-presenting cells, including DCs, macrophages, and, under inflammatory conditions, liver sinusoidal endothelial cells (137). Autoimmune hepatitis represents a paradigmatic example of dysregulated class II–mediated antigen presentation in the liver, characterized by aberrant activation of CD4+ T cells against hepatocellular antigens (138). In this setting, chronic inflammation drives progressive fibrosis, linking immune dysregulation directly to matrix stiffening. The resulting mechanical changes in the hepatic microenvironment may further amplify immune activation by altering DCs’ maturation, antigen uptake, and migration, thereby sustaining a vicious cycle of inflammation and fibrogenesis. For instance, matrix stiffness exerts negative regulation on tumor immunogenicity by inhibiting DC maturation and CD8+ T cell activation (139). In *in vitro* studies, matrix stiffness was also found to impair DC function by regulating cytokine secretion in immature and mature DCs (140) and limiting DC migration (141).

The relationship between matrix stiffness and antigen presentation is likely bidirectional. On one hand, fibrotic stiffening may impair effective antitumor immunity by restricting immune cell trafficking and promoting T-cell exhaustion (104). On the other hand, excessive or aberrant antigen presentation in a stiffened microenvironment may favor immune tolerance or autoimmunity, depending on the context and the dominant cytokine milieu. In autoimmune hepatitis, for example, persistent class II–restricted antigen presentation drives chronic T-cell activation and liver injury (138), whereas in HCC, tumor-associated antigen presentation often fails to elicit effective cytotoxic responses, instead contributing to immune exhaustion and escape (142). Kupffer cells and infiltrating macrophages are highly sensitive to mechanical cues and can adjust their antigen-presenting capacity in response to changes in tissue stiffness. In addition to regulating macrophage polarization, stiff matrices may skew macrophages toward phenotypes that preferentially induce Treg responses or tolerogenic signaling, thereby dampening antitumor immunity despite ongoing antigen presentation. Conversely, in autoimmune liver disease, similar mechanical cues may reinforce pro-inflammatory antigen presentation and perpetuate tissue damage (138, 143). These observations underscore the context-dependent nature of mechanoregulation in liver immunity. Under inflammatory conditions, HSCs can express class II molecules and costimulatory signals, contributing to local antigen presentation (144). Matrix stiffening enhances stellate cell activation and survival, potentially prolonging their immunomodulatory functions (52). In the fibrotic and cirrhotic liver, this may create microdomains in which antigen presentation, mechanical stress, and immune regulation converge to shape disease progression and carcinogenesis.

From a translational perspective, the interplay between matrix stiffness and antigen presentation critically influences immunotherapy in HCC. Matrix stiffness can compromise the efficacy of immune checkpoint inhibitors by impairing antigen processing and presentation, processes that are essential for reinvigorating exhausted T cells. Therapeutic normalization of tumor matrix stiffness harbors dual therapeutic potential in enhancing immune cell infiltration and restoring effective antigen recognition. At the same time, careful attention must be paid to the risk of unleashing autoreactive immune responses in a liver already predisposed to immune-mediated injury.

In conclusion, integrating mechanobiology with classical immunology reveals antigen presentation to be a key mechanistic axis via which ECM stiffness modulates liver immunity, autoimmunity, and hepatocarcinogenesis, thereby providing a more comprehensive framework for understanding and treating chronic liver disease.

## 6 The potential of matrix stiffness-targeted drugs in the treatment of HCC

Hepatic fibrosis, induced by disease-driven pathways such as inflammation and alcohol, is reversible. The alterations in the tumor mechanical microenvironment that are induced by increased matrix stiffness promote a positive feedback loop in the progression of hepatic fibrosis to HCC, and impede drug delivery and immunotherapy by forming a barrier (145). Therefore, the targeting of matrix stiffness within the TME could emerge as a pivotal strategy to enhance the efficacy of cancer treatment, and the relevant clinical trials have been summarized in **Table 1**.

**Table 1:** Clinical trials of targeting matrix stiffness-associated proteins in hepatic sclerosis diseases.

Trial ID	Drugs	Affected matrix stiffness protein	Conditions	Phase	Estimated or actual enrollment	Staus/results
NCT01858935	ND-L02-s0201 injection	HSP47	Healthy adults	Ib	56	Completed
NCT04099407	Prolonged-release pirfenidone	TGF-β	Advanced hepatic fibrosis	II	122	Favorable prognosis
NCT05115942	Hydronidone	TGF-β	HBV-injected hepatic fibrosis	III	248	Favorable prognosis
NCT01246986	Galunisertib	TGF-β	Advanced hepatocellular carcinoma	II	47	Favorable prognosis
NCT01707472	Simtuzumab	LOXL-2	HCV and HIV-infected adults	I	18	Well tolerated
NCT01672866/NCT0167289	Simtuzumab	LOXL-2	Cirrhosis caused by nonalcoholic steatohepatitis	IIb	219	Failure
NCT04857372	IAG933	YAP/TAZ	Solid tumors	I	156	Recruiting
NCT05789602	BPI-460372	YAP/TAZ	Solid tumors	I	82	Recruiting
NCT01139723	MINT1526A	Integrin α5β1	Hepatocellular carcinoma	I	24	Completed
NCT04194801	BLU-554	Fibroblast growth factor receptor	Hepatocellular carcinoma	Ib/II	18	Completed

### 6.1 Preclinical and clinically targeted ECM cross-linking therapy

#### 6.1.1 Targeted collagen crosslinking

Collagen is the most abundant protein in the TME, and its dynamic expression influences changes in matrix stiffness, as well as being closely associated with cancer development (146). Heat shock proteins (HSPs) contribute significantly to hepatic fibrosis by mediating protein folding, assembly, and stabilization, making them attractive therapeutic targets for matrix stiffness-directed interventions (147). Among these, HSP47 has emerged as a critical factor in collagen folding, secretion, and deposition, thereby promoting fibrosis progression (148). Yu et al. found that Biglycan (BGN), a proteoglycan, can directly interact with HSP47 to positively regulate ECM deposition and HSCs activation to increase liver stiffness (149). In a preclinical study, investigators developed a vitamin A-coupled liposomal siRNA formulation targeting HSP47 for selective delivery to HSCs in rats. After five treatment cycles, this approach achieved complete resolution of hepatic fibrosis and significantly prolonged survival in rats with fibrosis induced by CCL4 and other etiologies. The antifibrotic effects were dose-dependent and duration-dependent, highlighting the considerable potential of HSP47-targeted therapy for reversing hepatic fibrosis (150). In light of the preceding studies, a phase I double-blind randomised clinical trial was conducted to evaluate the safety, tolerability, and pharmacokinetics of vitamin A-coupled lipid nanoparticles ND-L02-s0201 injection containing siRNA against HSP47 in normal subjects. However, the antitumor effect of this drug in HCC requires further investigation.

Dysregulated activation of TGF-β is a hallmark of hepatic fibrosis (60). HSP47 is involved in regulating TGF-β-induced EMT and tissue matrix remodelling (151, 152). The TGF-β inhibitor pirfenidone is a small-molecule drug that can directly inhibit collagen production and has been found to indirectly inhibit collagen synthesis by inhibiting HSP47 expression (153). In a Phase II clinical trial involving 122 patients with advanced hepatic fibrosis who received an extended-release formulation of pirfenidone, 35% of treated patients achieved a significant reduction in fibrosis burden, in stark contrast to only 4.1% of patients in the control group (154). Hydronidone, a novel structural modification of pirfenidone, demonstrated significant antifibrotic efficacy in a phase III randomized controlled trial involving patients with chronic hepatitis B-related cirrhosis (155). *In vitro* and *in vivo* experimental findings have indicated pirfenidone’s efficacy in inhibiting HCC fibrosis progression and delaying cancer progression. Notably, as a PPARα agonist ligand, pirfenidone inhibits NF-κB p65 expression and nuclear translocation, thus hindering HCC progression and counteracting fibrosis (156). Pirfenidone controls HCC development by reversing DNA methylation (157). Besides, pirfenidone reduced HCC metastatic stiffness and enhanced the antitumor effect of sorafenib by inhibiting the CEMIP/TGF-β1/Smad signalling pathway (82). In addition, galunisertib, a TGF-β inhibitor, demonstrated superior efficacy in the Phase II treatment of advanced HCC (158). The present studies demonstrate the great potential of stiffness-targeted drugs in HCC prevention and combination therapy.

#### 6.1.2 Targeted LOX family

LOX participates in the key step of catalyzing collagen and elastin cross-linking in the ECM (159). Inhibition of LOX activity is considered to be an effective means of reducing matrix stiffness. Alba et al. used LOX inhibitors in multiple preclinical models of mouse cancers, including pancreatic, breast, and cholangiocarcinoma. These inhibitors effectively reversed tumor matrix stiffness and substantially improved survival rates in these models by enhancing effector CD8+ T cell accumulation in tumors when combined with PD-1 therapy (103). Exosomes miR-27b-3p can effectively slow down the degree of hepatic fibrosis by inhibiting LOXL2 (160). In a NASH model mouse, Auranofin reduced hepatic fibrosis and effectively improved survival by inhibiting LOX gene expression (161). β-Aminopropionitrile (BAPN) is a LOX-competitive inhibitor. In a CCL4-induced hepatic fibrosis mouse model, BAPN inhibited collagen cross-link accumulation and reduced matrix stiffness, effectively reversing hepatic fibrosis (162). Also, studies showed a marked decrease in collagen I after BAPN treatment in HCC mouse models (98). Furthermore, it can also inhibit HCC angiogenic tumor cell proliferation by reducing matrix rigidity (163). Lu et al. found that an increase in matrix stiffness was associated with a decrease in radiotherapy response, and verified that BAPN enhanced the radiotherapy efficacy of HCC cells by constructing ECM hydrogels containing Huh7, while effectively reducing matrix stiffness (164). Nevertheless, despite the evidence that BAPN exerts beneficial effects in the tumor treatment, the toxicity associated with its long-term administration poses a challenge for its practical clinical application.

Simtuzumab is the first humanised monoclonal antibody targeting LOXL2 and showed efficacy in animal studies. However, recent Phase 2 clinical trial results of Simtuzumab in hepatic fibrosis patients fell short of expectations (165). This unexpected result may be due to the drug concentrating only on LOXL-2, one of the LOX family homologues, resulting in insufficient antibody potency in the blood. Several potent dual LOX/LOXL2 and LOXL2/LOXL3 inhibitors have been developed, with pan-LOX family inhibitors being used in cancer therapy. For instance, PXS-5120A and PXS-5153A are both dual LOXL2/LOXL3 inhibitors administered orally, which have been shown to effectively reduce collagen content and cross-linking in hepatic fibrosis models, and to improve liver function (166). The pan-LOX family inhibitor PXS-5505 is safe in Phase I clinical studies, and its efficacy and safety are expected to be demonstrated in the next Phase I/IIa studies (167).

LOX is a copper-dependent amine oxidase found in the ECM (42). Therefore, LOX family activity can be indirectly targeted by limiting cellular copper utilisation. The copper chelator ammonium tetrathiomolybdate (ATTM) has simple administration and is well tolerated. Studies showed it has satisfactory ability to inhibit LOX family activity in pulmonary fibrosis mouse models (168). ATTM combined with hepatic artery ligation or lenvatinib treatment demonstrated good anti-tumor angiogenesis and anti-cancer effects in HCC (169, 170). These results demonstrate the potential for targeting LOX therapy to attenuate hepatic fibrosis and thereby slow HCC progression. Currently, clinical studies on direct LOX inhibitor application in HCC remain extremely limited. Further exploration of this field is warranted in the future.

### 6.2 Preclinical and clinical targeted signal transduction therapy

#### 6.2.1 Targeted YAP/TAZ (PROTAC) therapy

An alternative approach to targeting matrix stiffness involves suppressing ECM-derived matrix stiffness-induced mechanical signalling. As shown in previous studies, YAP/TAZ is a key mechanosensor in the Hippo pathway regulatory network. This pathway regulates liver regeneration and HCC progression (29, 171). High activity of Hippo pathway effectors YAP/TAZ impairs tumor drug delivery and induces stromal activation. Targeted disruption of stromal activation may enhance drug delivery efficiency (172). It is highlighted that YAP/TAZ may play an important role in HCC through stromal signalling. In addition, it was found that combining a YAP inhibitor with anti-PD-1 therapy significantly enhanced anti-PD-1 efficacy in HCC by regulating autophagy (173), the immunosuppressive microenvironment (174), and lipid metabolism (175). Kinases MST1 and MST2 (MST1/2) are core components of the Hippo pathway. Fan et al. developed XMU-MP-1, a small-molecule MST1/2 inhibitor that activates the downstream effector YAP and promotes cell and tissue repair and growth (176). In the same study, XMU-MP-1 was shown to increase liver regeneration rates in four acute and chronic liver injury mouse models, repair acetaminophen-induced injury, and prevent hepatic fibrosis in a chronic injury model (176). Compared with other complex therapies (e.g., biomaterials), it is more clinically valuable.

Verteporfin is a known inhibitor of the YAP, has been shown to disrupt the YAP-TEAD interaction, thereby inhibiting YAP-induced hepatomegaly and tumorigenesis (177). In addition, it has been shown to reverse YAP-induced sorafenib resistance (178). In HBsAg-positive mice, Verteporfin directly inhibited BMI-associated HCC development driven by YAP nuclear translocation (179). Wei et al. effectively blocked matrix stiffness-regulated HCC development *in vitro* by combining Verteporfin with the integrin inhibitor ATN-161 (84). Two clinical trials in solid tumors are also actively exploring the antitumor efficacy of YAP-TEAD interaction-disrupting drugs, with efficacy validated in preclinical studies (180, 181). Notably, YAP/TAZ act as transcriptional co-activators for STAT3 and AP-1 besides TEAD (182). Consequently, only inhibiting YAP/TAZ-TEAD interaction may not fully block their signalling activities. Proteolysis-targeting chimeras (PROTACs) are a promising approach for target protein degradation in cancer biomedical research, with high specificity and low toxicity (183). Notably, more than 50 PROTACs targeting proteins have now been successfully developed, with some currently in clinical trials for efficacy evaluation. For example, SD-36, a STAT3-targeting PROTAC developed by Wang et al., showed superior STAT3 selectivity and effective anti-tumor effects in cancers (184, 185). The novel PROTAC SIRT6 degraders exhibited superior efficacy in inhibiting HCC progression compared to parental inhibitors, and showed enhanced efficacy when combined with radiosensitisation and sorafenib (186). Therefore, it can be hypothesised that target proteins may be degradable via PROTAC technology, which effectively inhibits YAP/TAZ expression. In addition, common drugs such as metformin, statins, and angiotensin inhibitors indirectly regulate YAP/TAZ activity (29).

#### 6.2.2 Integrins, FAK, and other targeted therapies

Integrins are cell surface proteins connecting the ECM and actin skeleton, transmitting mechanical signals intracellularly by sensing matrix stiffness changes. They mediate downstream responses such as cell adhesion, migration, and signal transduction (187). Abnormal integrin activation promotes a range of adverse outcomes, including HCC growth, proliferation, metastasis, and chemoresistance (188, 189). Therefore, targeting integrins is a key strategy for blocking the matrix stiffness signalling pathway. Synstatin (SSTN92-119) inhibited HCC angiogenesis and proliferation by interfering with the CD138/integrin αVβ3 interaction (190). Ke et al. prepared a monoclonal antibody (CD151 mAb 9B) against the CD151/integrin α6β1 binding site, which effectively blocked tumor progression in HCC mice (188). Fucoidan is a naturally occurring sulfated polysaccharide that is found in brown algae. It impedes tumor metastasis in various HCC cells by targeting integrin αVβ3 and related signalling pathways, with validation *in vivo* (191). Moreover, the targeting of integrin-related molecules such as FAK and PI3K has the capacity to regulate integrin signalling indirectly. Tan et al. synthesized 5-fluoro-7H-pyrrolo[2, 3-d]pyrimidine derivatives as FAK inhibitor scaffolds based on the FAK-PF-562271 co-crystal complex. *In vivo* experiments showed that these derivatives potently inhibited HCC malignant proliferation (192). The FAK inhibitor VS4718 was found to reduce hepatic fibrosis and enhance the efficacy of immunotherapy in a mouse model of HCC (193). Wortmannin is a PI3K inhibitor. Recent findings have shown the potential of this substance to impede the growth of tumors in HCC, with this effect being more pronounced in conjunction with sorafenib treatment (194). Despite extensive preclinical research on integrin targeting, results from a Phase I trial evaluating the safety, efficacy, and tolerability of MINT1526A (RG-7594), a monoclonal antibody targeting integrin α5β1, in patients with various solid tumors (including HCC) failed to demonstrate the expected clinical activity (195). Similarly, clinical trial results for integrin-targeted agents in other tumor types have been somewhat unsatisfactory. This disappointing outcome is likely attributable to the extensive and heterogeneous *in vivo* expression of integrins. Therefore, the development of highly specific integrin-targeted oncolytic agents remains a key research endeavor.

### 6.3 Targeting matrix stiffness improves immunotherapy efficacy

The advent of immunotherapy has precipitated a paradigm shift in cancer treatment. Notably, single-agent immunotherapy has proven ineffective for a significant proportion of HCC patients. This is likely due to elevated tumor tissue stiffness, which impairs drug delivery and inhibits the infiltration of anti-tumor immune cells. For example, shear wave elastography (SWE)-based detection of HCC tumor stiffness revealed that hepatic matrix stiffness serves as a predictive marker for the therapeutic efficacy of PD-1 inhibitor-lenvatinib combination therapy (196). ICIs exhibit impaired efficacy in cirrhotic-associated HCC, as this cancer subtype induces the formation of neutrophil extracellular traps (NETs) that generate an immunosuppressive TME (197). Liu et al. found that SPP1^+^ macrophages interact with CAFs to induce ECM remodelling and drive the formation of a tumor immune barrier, thereby attenuating anti-PD-1 therapeutic efficacy in HCC; conversely, SPP1 inhibition restores immunotherapeutic sensitivity (198). A similar effect was observed in an HCC mouse model, in which TGF-β neutralizing antibody-mediated amelioration of the tumor fibrotic microenvironment promoted the redistribution of CD8+ T cells into the tumor parenchyma (104). Cai et al. observed that immune escape was more pronounced in HCC patients with CTNNB1 mutations, and that MMP9-targeted therapy restored CD8+ T cell-mediated anti-tumor immunity and enhanced anti-PD-1 efficacy in these patients (199). These preclinical findings provide robust evidence for developing matrix stiffness-targeted agents for the combination of antifibrotic therapy and immunotherapy in HCC. A Phase Ib/II study was conducted to assess the safety and efficacy of combining the fibroblast growth factor receptor 4 (FGFR4) inhibitor BLU-554 with an anti-PD-L1 monoclonal antibody in patients with locally advanced or metastatic HCC. The trial concluded that this combination therapy shows considerable promise for clinical translation (200).

CAR-T therapy, an autologous T cell therapy involving the harvesting of a patient’s autologous immune cells and genetic engineering of T cells to express chimeric antigen receptors (CARs) on their surface, represents a leading modality of adoptive cell therapy (ACT) (201). However, CAR-T cells’ ability to penetrate tumor tissues is easily limited by the dense ECM-derived barrier, leading to failed antigen clearance and eventual CAR-T cell depletion (202). Wang et al. specifically inhibited tumor growth with minimal to no toxicity by utilizing CAR-T cells targeting the CAF marker FAP (203). A novel immunotherapeutic strategy targeting ECM laminin degradation effectively reduces tumor stiffness while simultaneously enhancing tumor perfusion and survival in murine models (204). However, targeting fibrotic sites while preserving the ECM structure of normal tissues remains a major hurdle. In recent years, vaccination-based immunocoupling therapy has emerged as a new therapeutic strategy. FN-targeted immunocouplers selectively destroy tumor cells and blood vessels by binding to the stroma, exhibiting site-specific extravasation, while minimal extravasation occurs in normal blood vessels, thereby protecting normal tissues (205). In addition, vaccination-based design of specific immunotherapies against fibrotic cells shows potential for immunotherapy of cirrhotic HCC (206).

The application of nanomaterials in cancer therapy has become a major hotspot in current research and has shown great potential in enhancing immunotherapy. MMP-sensitive nanocarriers effectively promote degradation of the physical barrier induced by matrix stiffness, thereby enhancing the therapeutic efficacy of targeted immunotherapeutics delivered by the carriers and potently inhibiting HCC growth in murine models (207). Given the crucial role of the ROCK pathway in regulating matrix stiffness, Shen et al. developed a novel RGD-modified liposome (RGD@LP-Y) to deliver the ROCK inhibitor Y-27632 to HCC tumor sites. By remodeling tumor matrix stiffness, this strategy significantly enhanced the therapeutic efficacy of radiotherapy-immunotherapy combination for HCC (208). Photodynamic therapy (PDT) mediated by semiconducting polymer-based ECM nanoremodelers (SPNcb) further inhibits tumor growth and metastasis by releasing LOX inhibitors upon recognition by tumor markers, thereby reducing tumor matrix stiffness and acting in synergy with PD-L1 immunotherapy (209). Studies have reported that photothermal therapy (PTT) can reverse tumor matrix stiffness and potently inhibit tumor progression (210). Yang et al. utilized a tandem photothermal-chemo-immunotherapy triple synergistic strategy for HCC, combining nanocarriers, sorafenib, and an anti-PD-L1 antibody, to overcome tumor immune evasion, improve the immunotherapeutic response rate, and achieve complete remission in an orthotopic HCC mouse model (211).

## 7 Conclusions and future directions

Hepatic fibrosis is a prevalent precursor of HCC. During this process, ECM-associated proteins like collagen undergo excessive deposition and crosslinking, increasing hepatic microenvironmental stiffness. Mechanosensors then transduce mechanical signals into cells, inducing phenotypic changes in hepatic and stromal cells. Meanwhile, cells secrete molecules including MMPs that drive ECM stiffness degradation, rendering ECM stiffness dynamic throughout tumorigenesis and thereby eliciting a cascade of downstream effects that regulate tumor cell proliferation, migration, chemoresistance and immune evasion (20). Therefore, accurate detection of liver stiffness across different HCC stages is critical for assessing patient prognosis and guiding targeted therapy. Although physical examination, color Doppler ultrasound, SWE, and transient elastography are used clinically, each method has inherent limitations. Thus, there is an urgent need to develop more accurate and non-invasive detection modalities. In addition, ECM-associated proteins have the potential to serve as biomarkers for predicting the response to HCC matrix stiffness-targeted and immunotherapies, and also represent a future approach for assessing liver stiffness.

Many preclinical studies have shown great potential of matrix stiffness-targeted therapies in cancer. A primary strategy directly targets collagen degradation to reduce matrix stiffness and enhance tumor drug sensitivity. Another indirectly reduces matrix stiffness by inhibiting collagen synthesis and assembly, and blocking mechanotransduction signals. Some of these strategies have been successfully translated into clinical trials with encouraging outcomes. However, others (e.g., integrin inhibitors) have yielded discouraging results, highlighting the need for further research into the specific molecular mechanisms of matrix stiffness in HCC to identify key targets for reversing it.

Growing numbers of studies are focusing on the critical role of stromal stiffness in cancer immunotherapy.il The therapeutic strategy of targeted matrix stiffness combined with immunotherapy has emerged as a novel direction in cancer treatment and has achieved notable progress. However, numerous limitations persist in the clinical translation and practical application of this strategy, which may explain why clinical research into matrix stiffness-targeted immunotherapy for HCC remains extremely limited to date. First, although it has been well established that diverse immune cells (including macrophages and T cells) exhibit mechanosensitivity to matrix stiffness, the precise mechanosensors and downstream signaling pathways may vary across distinct immune cell subtypes. For example, stiffness-induced Treg differentiation via Piezo1 (111) may occur independently of the pathways that regulate cytotoxic CD8+ T cell exhaustion (116). Further investigation into the specific cell types impacted using techniques such as single-cell sequencing is required to mitigate unintended immunomodulatory effects induced by matrix stiffness-targeted drugs. Second, the temporal relationship between hepatic fibrosis reversal and immune reactivation presents therapeutic challenges. While reducing tissue matrix stiffness enhances drug delivery and immune cell infiltration, rapid degradation of rigid ECM may suddenly expose previously sequestered neoantigens or alter chemotactic gradients, which may trigger excessive or aberrant immune responses. Therefore, preclinical models enabling real-time monitoring of matrix stiffness changes, immune cell migration, and tumor dynamics are required to identify an optimal “therapeutic window” for stiffness-targeted combination therapy. Within this window, hepatic fibrosis reversal can promote rather than interfere with effective anti-tumor immunity. Finally, manipulating the liver matrix stiffness, the liver being a classic tolerogenic organ, carries inherent risks. Excessive softening of the liver matrix may not only promote the infiltration of anti-tumor immune cells but also disrupt the physiological barriers that maintain peripheral tolerance to enteric antigens. This may potentially trigger autoimmune-like hepatitis or exacerbate the underlying inflammatory state of the organism. Moreover, since the stiffness of the liver ECM provides essential mechanical support for liver tissue structure and regeneration, its excessive reduction may impair the liver’s normal repair capacity following resection or damage induced by radiotherapy and chemotherapy. In conclusion, future therapeutic strategies must prioritize spatial and temporal precision through the development of novel stimulus-responsive biomaterials, cell-specific delivery systems, and pharmacologically modifiable agents. This strategy is designed to correct pathological liver matrix stiffness without disrupting the mechanical baseline essential for maintaining liver homeostasis and facilitating tissue repair, thereby enhancing the efficacy of immunotherapy for patients with HCC.

## Glossary

HCC: Hepatocellular carcinomas
ECM: Extracellular matrix
TME: Tumor microenvironment
CAF: Cancer-associated fibroblast
ICI: Immune checkpoint inhibitor
TKI: Tyrosine kinase inhibitors
GAG: Glycosaminoglycan
MMP: Matrix metalloproteinase
TIMP: Tissue inhibitor of metalloproteinase
HSC: Hepatic stellate cell
FN: Ffibronectin
ROCK: Rho-associated kinase
PG: Proteoglycan
SDC1: Syndecan-1
TGF-β: Transforming growth factor β
NMJ: Neuromuscular junction
YAP: Yes-associated protein
dlSEC: Degrading liver sinusoidal endothelial cell
LH: Lysyl hydroxylase
PLOD2: 2-Oxoglutarate 5-Dioxygenase 2
LOX: Lysyl Oxidase
LOXL: Lysyl Oxidase-like
LSEC: Liver sinusoidal endothelial cell
RhoA: Ras homolog gene family member
COL1A1: Collagen type I alpha 1
COL3A1: Collagen type III alpha 1
CLCF1: Cardiotrophin-like cytokine factor 1
LCSC: Liver cancer stem cell
EGFR: Epidermal growth factor receptor
EMT: Epithelial-mesenchymal transition
CXCR4: C-X-C chemokine receptor type 4
VEGF: Vascular endothelial growth factor
AMP: Adenosine monophosphate
AMPK: Adenosine monophosphate -activated protein kinase
SCD1: Stearyoyl-CoA desaturase 1
ACADL: Long-chain acyl-CoA dehydrogenase
DC: Dendritic cells
CTL: Cytotoxic T cells
Treg: Regulatory T cell
TAM: Tumor-associated macrophage
NK: Natural killer cell
TEX: T-cell exhaustion
TF: Transcription factor
Osr2: Odd-skipped related 2
TNF-α: Tumor necrosis factor-α
MMT: Macrophage-to-myofibroblast transition
α-SMA: α-smooth muscle actin
HSP: Heat shock proteins
BGN: Biglycan
BAPN: β-Aminopropionitrile
ATTM: Ammonium tetrathiomolybdate
MST1/2: Kinases MST1 and MST2
PROTAC: Proteolysis-targeting chimera
SWE: Shear wave elastography
NET: Neutrophil extracellular trap
CAR: Chimeric antigen receptor
MHC: Major histocompatibility complex
